# Ecofriendly Long Life Nanocomposite Sensors for Determination of Carbachol in Presence of Choline: Application in Ophthalmic Solutions and Biological Fluids

**DOI:** 10.3390/s19102357

**Published:** 2019-05-22

**Authors:** Eman A. Al-Harbi, Mona H. Abdelrahman, Amira M. El-Kosasy

**Affiliations:** 1 Department of Chemistry, Faculty of Science, Taibah University, Al-Madina Al-Mounawara 42353, Saudi Arabia; 2 Department of Pharmaceutical Analytical Chemistry, Faculty of Pharmacy, Ain Shams University, Cairo 11566, Egypt; monahamdyph@yahoo.com (M.H.A.); kosasy2030@yahoo.com (A.M.E.-K.)

**Keywords:** graphene, carbachol, sensor, nanocomposites, ecofriendly

## Abstract

Several emerging nano scale forms of carbon are showing great promise in electrochemical sensing such as graphene and multi-walled carbon nanotubes (MWCNTs). Herein we present an ecofriendly method to fabricate long life and sensitive ion selective sensors based on graphene and MWCNTs nanocomposites with no need for volatile organic solvents. Both sensors were fabricated, for the analysis of carbachol in ophthalmic solutions, plasma and urine where ion- association complex was formed between cationic carbachol and anionic Sodium tetra phenyl borate (NaTBP) in a ratio 1:1. Both sensors were evaluated according to the IUPAC recommendation data, revealing linear response in the concentration range 10^−7^ M to 10^−2^ M with near Nernstian slopes 50.80 ± 5 and 58.14 ± 3 mV/decade and correlation coefficients 0.9992 and 0.9998 for graphene and MWCNTs based sensors, respectively. Both sensors were successfully applied as stability indicating method for the analysis of carbachol in presence of its metabolite choline, in ophthalmic preparations, in plasma and urine showing good recovery percentage values. MWCNTs based sensor showed some advantages over graphene sensor regarding lower limit of detection (LOD), longer life time and higher selectivity towards carbachol. Statistical comparison of the proposed sensors with the official method showed no significant difference for accuracy and precision.

## 1. Introduction

Both graphene and carbon nanotubes are considered to be excellent candidates as second-phase fillers in composite materials as they have exceptional electrical conductivity [1]. Graphene has been the subject of extensive scientific research since its discovery was reported in 2004 in the journal *Science*, outlining the isolation of a single atomic layer of graphite [2,3,4]. It completes the family of different carbon allotropes that are stable at ambient conditions and is considered as the thinnest, most flexible material known. Graphene is a two-dimensional (2D) sheet of carbon atoms bonded by sp^2^ bonds. It is a fantastic material for use in electrochemical analysis, owing to the combination of its interesting chemical properties and its unique assortment of physicochemical properties [5,6,7,8].

This configuration provides this material with extraordinary properties, such as large surface area, excellent thermal and electrical conductivity, and high electron mobility at room temperature. Graphene is also considered to be a zero-gap semiconductor, so it can be considered as a metal [9].

**Figure 1:**
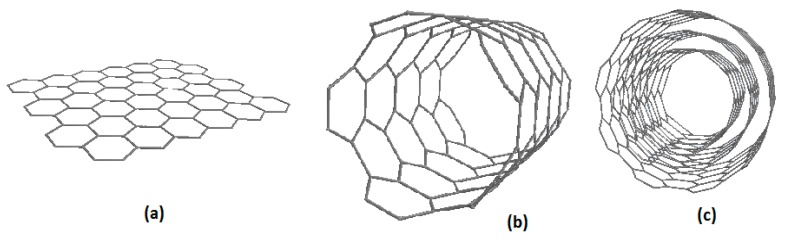
Molecular models of sp^2^-bonded carbon nanostructures: (**a**) graphene clusters, (**b**) single-walled carbon nanotubes (SWCNTs), (**c**) multiwalled carbon nanotubes (MWCNTs) formed from continuous concentric graphene tubes.

The earliest recognized synthesis of a multiwalled carbon nanotube (MWCNT) was in 1952 by Radushkevich and Lukyanovich [10]. Carbon nanotubes (CNT) are considered as graphene layers rolled into cylinders that consist of a planar hexagonal arrangement of carbon–carbon bonds [11]. Due to their unique structure, CNTs have very promising physicochemical properties, such as large surface area, high rigidity, high tensile strength that is nearly 50 times higher than steel, high electrical conductivity, high thermal conductivity, and low density (Figure 1) [12]. They are chemically stable and considered to be better conductors than copper. All these characteristics make CNTs unique materials with the potential for a lot of applications. It is worth noting that MWCNTs are often preferred over their single-walled counterparts due to the lower cost of production and rapid response [13].

Due to their outstanding mechanical, electrical, chemical, and thermal properties, carbon nanostructures have recently found application in many areas, including electronics, composite materials, and sensors [14,15,16,17].

**Figure 2:**
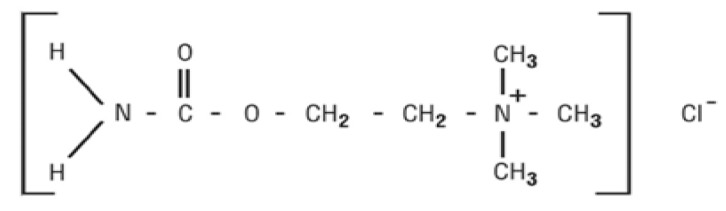
The chemical structure of carbachol chloride.

Carbachol (2-carbamoyloxyethyl(trimethyl) azanium; chloride) is a choline ester and a positively charged quaternary ammonium compound with pKa (negative log of the acid dissociation constant) equal to 15.23. It is used for glaucoma treatment or ophthalmic surgery (Figure 2) [18]. It is a potent cholinergic agent that constricts the iris and ciliary body, resulting in a reduction of intraocular pressure in patients with glaucoma. Moreover, carbachol is used in the treatment of postoperative intestinal atony and postoperative retention of urine, for which it is given by subcutaneous injection or by mouth [19]. It is also used to stop supraventricular paroxysmal tachycardia when all other measures have failed [20]. For treatment of glaucoma, the concentration and frequency of instillation of the carbachol ophthalmic solution must be adjusted to the requirements and responses of individual patients as determined by tonometric readings before and during therapy. Decreased concentrations of carbachol in an ophthalmic formulation may prevent effective decrease of intraocular pressure, which may have deleterious impact, such as iris prolapse. Also, carbachol undergoes degradation in alkaline solutions, thus producing choline, which is also a product of carbachol metabolism. Intoxications with carbachol has previously been reported in the literature [21,22,23,24]. Investigations have also found that oral intoxications with carbachol can cause nausea, sweating, hypotension, bradycardia, and severe persistent cholinergic symptoms, leading to a case of acute cardiovascular failure and, finally, fatal poisoning [25].

Therefore, in toxicity cases, a method to selectively analyze carbachol in ophthalmic solutions, in presence of its degradation product, and in urine and plasma is required. Moreover, analytical methods are needed to ensure that the concentration of carbachol in pharmaceutical preparations remain at therapeutically active levels. A review of the literature shows that several methods have been developed for the determination of carbachol, including fluorescence, proton magnetic resonance spectroscopy, potentiometry, indirect UV-VIS spectrophotometry, IR spectroscopy, ion chromatography, and the official colorimetric method [26,27,28,29,30,31,32].

Among electrochemical sensors, potentiometric sensors are considered as an important class, which transduces the activity of a certain analyte dissolved in a solution into an electrical potential [33]. To the best of our knowledge, the only electrochemical method published in the literature for the determination of carbachol in ophthalmic preparation was done by Jeffrey et al. [28]. The method utilized 2-nitrophenyl octyl ether (NPOE) as the plasticizer and tetrahydrofuran (THF) as the organic solvent, and the stability times of the investigated sensors were shorter than our sensors. Moreover, the published electrochemical method was applied only on ophthalmic preparation and not in biological matrices.

In our proposed method, we demonstrate for the first time eco-friendly, long-life potentiometric nanocomposite sensors for the accurate, precise, and selective determination of carbachol in ophthalmic solutions, in presence of choline, and in biological fluids. The proposed sensors can be considered as cheap, easy, and fast tools for monitoring urine and serum concentration of carbachol in cases of oral intoxication without the need for tedious and time-consuming extraction methods. The effect of incorporating nanoscale particles on potential stability, considering response time and shelf life of the proposed sensors, was found to be superior to ordinary scale sensors.

## 2. Materials and Methods

### 2.1. Apparatus

Ag/AgCl double-junction was used as external reference electrode (Thermo Scientific Orion 900,200 (Chelmsford, UK); 3.0 M KCl saturated with AgCl as an inner filling solution and 10% KNO_3_ as a bridge electrolyte). Jenway digital ion analyzer (model 3330; Essex, UK) was used for potentiometric measurements. pH glass electrode no. 924005-BO3-Q11C (Essex, UK) was used for pH adjustment. A Bandelin Sonorex magnetic stirrer and heater, model Rx 510 S (Budapest, Hungary), were used.

### 2.2. Chemicals and Reagents

All chemicals and reagents used in this study were of analytical grade, and water was bidistilled. Graphene nanoplatelets (particle size 25 µm, thickness 6–8 nm, bulk density 0.03–0.1 g/cm^3^), MWCNTs (>95% carbon basis, diameter 50–90 nm, bulk density 0.007 g/cm^3^), choline, carbachol, sodium tetraphenylborate (NaTPB) were purchased from Sigma Aldrich (Steinheim, Germany). Urea, 2-chloroethanol, potassium carbamate, triethylamine, sodium chloride, potassium chloride, calcium chloride hydrate, paraffin oil, and benzalkonium chloride were purchased from El Nasr Company (Cairo, Egypt). Miostat Intraocular Solution (labeled amount of 0.01% carbachol), manufactured by Alcon Laboratories, INC. (Fort Worth, TX, USA), was obtained from the local market. Jestryl ampoules (labeled amount 0.00025 g carbachol/mL) manufactured by veb ankerwerk Rudol stadt thur chem. pharm fabric (Rudolstadt, Germany) was obtained from the local market.

### 2.3. Stock Standard Solution

Carbachol fresh stock solution (1 × 10^−2^ M) was prepared by dissolving 0.1826 g of carbachol in bidistilled water and then filling to the volume of 100 mL using bidistilled water. Working solutions starting from 1 × 10^−7^ to 1 × 10^−3^ M were prepared by suitable dilution from the fresh stock solution (1 × 10^−2^ M) using bidistilled water.

### 2.4. Procedures

#### 2.4.1. Preparation of the Ion-Association Complex

The ion-association complex was prepared by mixing 100 mL of carbachol chloride 10^−3^ M working solution with 100 mL of a saturated NaTPB aqueous solution containing 0.342 g of NaTPB. The resulting white precipitate was filtered, then washed with water, dried at room temperature, and ground to obtain a fine powder, which was used for the fabrication of both sensors.

#### 2.4.2. Sensor Fabrication

In a mortar, 20 mg ion-association complex and 300 mg graphene and MWCNTs for sensors 1 and 2, respectively, were mixed and homogenized into a smooth paste while using paraffin oil as binder in the ratio 60%:40% *w*/*w* (carbon material: oil). The paste was firmly packed into a plastic tube cavity with a diameter of 3.5 mm with extreme care to prevent formation of air gaps. A copper wire with a diameter of 1 mm was inserted into the other end to obtain appropriate electrical contact. After assembly, the sensors were finally conditioned by soaking each one separately in 10^−2^ M carbachol solution overnight before measurements.

#### 2.4.3. Sensor Calibration

The calibration of the proposed sensors was achieved by separately transferring 40-mL aliquots of carbachol throughout the concentration range (1 × 10^−7^ to 1 × 10^−2^ M) into six 100-mL beakers. The measured electromotive force (emf) values within ± 5 mV and ± 3 mV were recorded for sensors 1 and 2, respectively. Washing was done between measurements using bidistilled water for both sensors. Calibration graphs were obtained by recording emf values against negative logarithmic carbachol concentration, and the regression equation for each sensor was computed for the linear part of the curve.

#### 2.4.4. Application in Ophthalmic Solutions

The content of 10 vials (8.25 × 10^−4^ M) of Miostat and 10 ampoules (1.38 × 10^−3^ M) of Jestryl were quantitatively transferred separately into a 100-mL volumetric flask and filled to the mark with bidistilled water. Suitable dilutions were prepared for each volumetric flask to obtain concentration of 8.25 × 10^−6^ M of Miostat and 1.38 × 10^−6^ M of Jestryl. The recovery values of carbachol using the fabricated sensors were determined from the corresponding regression equations.

#### 2.4.5. Application in Biological Fluids

The determination of carbachol in spiked human plasma and urine samples were separately achieved by preparing suitable dilution of working standard solutions. The sensors were immersed separately in the prepared biological samples, and the emf was recorded each time. Washing was done between measurements using bidistilled water for both sensors. The emf values produced were recorded, and the concentrations were calculated from previous regression equations. Results obtained were expressed as recovery % ± SD.

#### 2.4.6. Determination of Carbachol in Presence of Choline

Ten milliliters of 10^−3^ M carbachol working solution was transferred separately into nine 100-mL volumetric flasks. Variable amounts of choline were added starting from 10% to 90%, and each flask was filled to 100 mL with bidistilled water. The final concentration of carbachol solution in the nine volumetric flasks was 10^−4^ M carbachol.

## 3. Results

### 3.1. Sensor Mechanism and Fabrication

**Figure 3:**
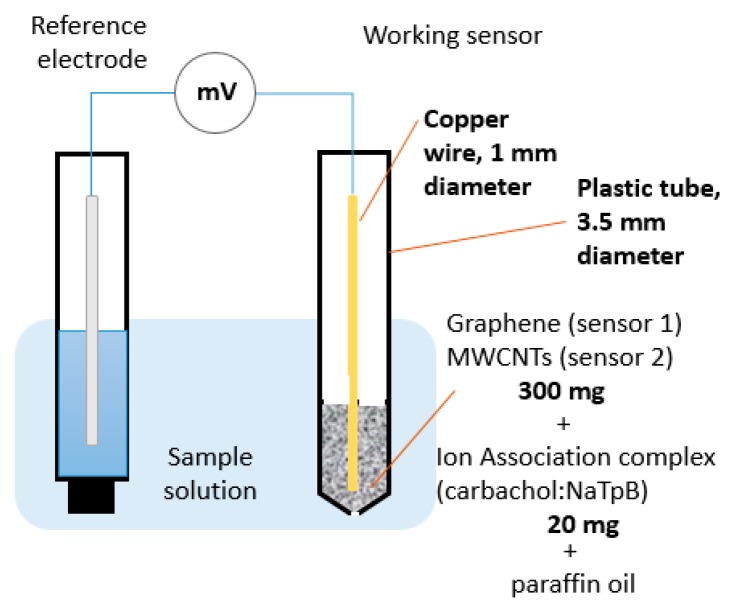
Experimental setup of the proposed sensors.

The experimental setup of the proposed sensors is illustrated in Figure 3. The sensing part of the proposed sensors was the carbon paste formed from incorporating ion-association complex of cationic carbachol and anionic NaTPB in the ratio of 1:1, along with nanoscale carbon materials and paraffin oil as binding agent. The positively charged quaternary ammonium group was found to have a high tendency to form an ion-pair complex with the negative borate ion. A potential developed across the interface between the carbon paste and the carbachol aqueous solution, which was concentration-dependent. The potential difference was measured between the proposed sensors separately and the reference electrode, and the value measured was directly related to −log [molar concentration of carbachol] according to the Nernst equation [34]. Slope values of the proposed sensors were about 60 mV/decade, which is the typical value for monovalent substances like carbachol, which has a quaternary ammonium group. The typical amount of binder added should be 15–42% *w*/*w* [35]. In this study, we found that adding paraffin oil to carbon nanomaterials in the ratio of 40%:60% *w*/*w* gave the most mechanically robust and stable paste. Both graphene and MWCNTs have large surface area and excellent thermal and electrical conductivity compared to conventional graphite. In our proposed sensors, there was no need for using hazardous plasticizer or volatile organic solvents because paraffin oil is sufficient to make a homogenous paste. As a result, the greenness of the method was enhanced. Solvent-free methods are considered to be the greenest option for any analytical method [36]. Most efforts in making chemical processes greener emphasize the need for using safer, less toxic, and more benign solvents, or the elimination of solvents altogether, and reduction in the use of reagents and auxiliaries.

The electrochemical cell assembly of the proposed sensors can be illustrated diagrammatically as follows:

For sensor 1:

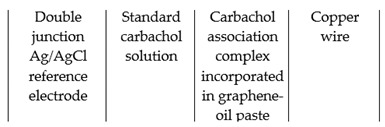



For sensor 2:

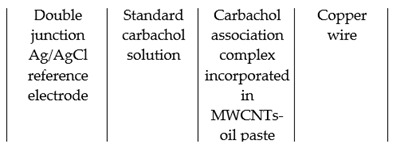



### 3.2. Sensor Calibration

**Figure 4:**
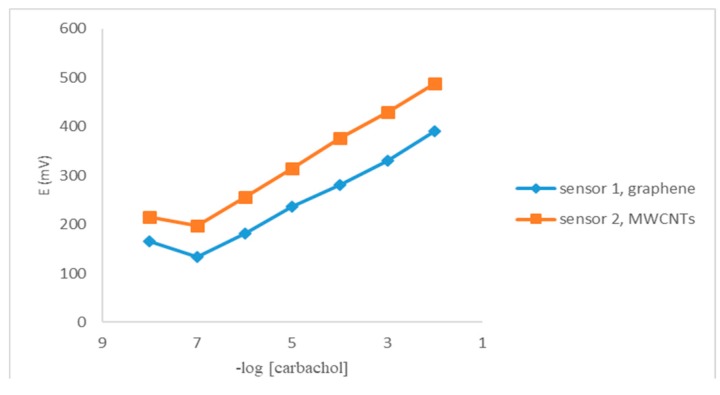
Profile of the potential in mV versus −log [carbachol] using the proposed sensors.

The obtained Nernstian slopes of the calibration plots were 50.80 and 58.14 mV/decade for sensors 1 and 2, respectively (Figure 4). The potential exhibited by the proposed sensors for constructive measurements of standard carbachol solution in the same day and from day-to-day showed RSD% values between 0.11% and 2.1%. Calibration slopes did not change by more than ± 5 and ± 3 mV/decade concentration over a period of 45 and 60 days for sensors 1 and 2, respectively.

### 3.3. Performance Characteristics of the Proposed Sensors

The electrochemical performance of the fabricated sensors was achieved according to the International Union of Pure and Applied Chemistry (IUPAC) recommendation data [37] and are summarized in Table 1. Calculation of limits of detection was easily achieved from the extrapolated linear segments of the calibration graphs, and they demonstrated low LOD values. Fresh new surface of the fabricated paste could be easily obtained by expelling an excess of the paste and gently polishing the surface onto soft paper. The long stability time of the proposed sensors was due to surface renewability, with the surface being renewed every 1.5 months and 2 months for sensors 1 and 2, respectively. The new surface obtained gave the same accurate and precise results, doing this 3–4 times for both sensors could extend the lifetime to 6 months and 8 months for sensors 1 and 2, respectively. Our eco-friendly, organic, solvent-free proposed sensors also showed excellent correlation coefficients. Organic solvents that are commonly used in fabricating sensors, such as THF [28,38,39], are considered as volatile organic compounds (VOCs), which are irritants and are flammable, thereby causing adverse health effects such as eye irritation and headaches. VOCs are also a hazardous environmental concern as they can form low-level ozone through free radicals or oxidation processes [40]. Regarding our proposed sensors, the MWCNT-based sensor had longer lifetime, better Nernstian slope, better recovery, and more precision and selectivity than the graphene-based sensor.

### 3.4. Effect of Nanoscale Carbon Materials

Fabrication of the proposed sensors using graphene and MWCNTs as electrode materials had a great, pronounced effect on sensitivity, stability, and the response time compared to ordinary scale-based sensors. A comparison between the proposed sensors and sensors investigated by the published ordinary scale method [28] is shown in Table 2. The mechanism of action is owed to the fast ion-to-electron transition offered by graphene and MWCNTs as nanoscale carbon materials, which allowed short response times for both sensors. Moreover, the lifetime was 1.5 and 2 months for sensors 1 and 2, respectively. Being excellent electrical conductors [1], nanoscale carbon materials can act as boosters to enhance the rate of electron transfer of electroactive species (ion-association complex). They also significantly improve the surface-to-volume ratio, which results in improved accumulation of carbachol molecules on the sensor surface and thus increase the sensitivity and selectivity.

### 3.5. pH Study

**Figure 5:**
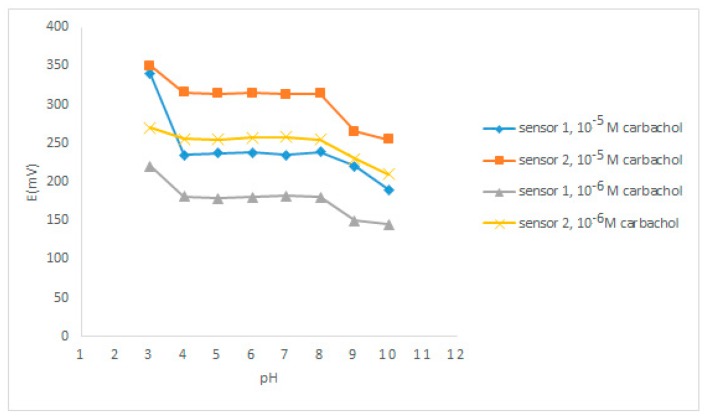
Effect of pH on the response of the proposed sensors.

Two different concentrations were used to study the effect of pH on both sensors. 10^−5^ M and 10^−6^ M carbachol working solutions were used over the pH range 3–10 using diluted solutions of HCl and NaOH. The results showed that both sensors exhibited constant potentials over pH range 4–8, as shown in Figure 5. Therefore, this range was used as a working pH range for the fabricated sensors. Results showed that, above pH 8, the potentials became lower, which may be caused by the instability of carbachol at alkaline pH and its degradation to choline.

### 3.6. Sensor Selectivity

The separate solution method was used to get the potentiometric selectivity coefficients of the proposed sensors using the following rearranged Nikolsky–Eisenman equation [41]:

Log Kpot _A, B_ = [(E_B_ − E_A_)/S] + (1 − Z_A_/Z_B_)] Log [A], where E_A_ is the electrode potential of 10^−4^ M carbachol solution, E_B_ is the electrode potential of 10^−4^ M solution of the interferent ion, and S is the slope of the calibration curve. Commercially available ophthalmic solutions of carbachol were found to contain sodium chloride, potassium chloride, calcium chloride, sodium acetate, sodium citrate, and benzalkonium chloride. The effect of these interferents on the response of the proposed sensors was studied. Inorganic cations, such as Na^+^, K^+^, and Ca^+2^ were studied. These ions can be found in plasma and urine besides ophthalmic solutions. The influence of benzalkonium chloride as interferent was investigated as it is used as a preservative and wetting agent in pharmaceutical preparation. The effect of 2-chloroethanol, urea, triethylamine, and potassium carbamate were also investigated as they are precursors used during carbachol synthesis. The results of the selectivity coefficients, as shown in Table 3, indicated excellent selectivity of the proposed sensors toward carbachol with no significant interference from all possible interferents, especially with the structurally related compound choline as its calculated log K values were 0.7 × 10^−3^ and 0.2 × 10^−4^ for sensors 1 and 2, respectively.

### 3.7. Application in Ophthalmic Preparation and Spiked Biological Samples

The determination of carbachol using the proposed sensors was successfully achieved in Miostat and Jestryl pharmaceutical solutions, and the results showed excellent recoveries between 99.74% and 100.98%, as shown in Table 4. The proposed sensors were also applied for the determination of carbachol in spiked plasma with mean recovery values of 101.6 ± 2.68 and 99.70 ± 0.23 for sensors 1 and 2, respectively, and in urine samples with mean recovery values of 104.8 ± 4.76 and 99.7 ± 0.25 for sensors 1 and 2, respectively, without pretreatment. Accurate and precise recoveries were obtained, as shown in Table 5. The results showed minimal interference from plasma and urine complex matrices.

### 3.8. Determination of Carbachol in Presence of Choline as a Stability Indicating Method

The results of recovery of carbachol in presence of increasing concentrations of choline showed the excellent selectivity of the method. The mean recovery percentages of carbachol in presence of increasing percentage of its degradate (10%–90%) were 101.70 and 101.12 for sensors 1 and 2, respectively. The detailed results are summarized in Table 6.

### 3.9. Statistical Comparison

The results of the fabricated sensors were statistically compared with results of the official pharmacopoeial method [32] using the student’s *t*-test and F-test at *p*-value equal to 0.05, as shown in Table 7. The calculated *t* and F values were less than the critical values, indicating that there was no significant difference between the mean and variances of both methods.

## 4. Discussion

**Figure 6:**
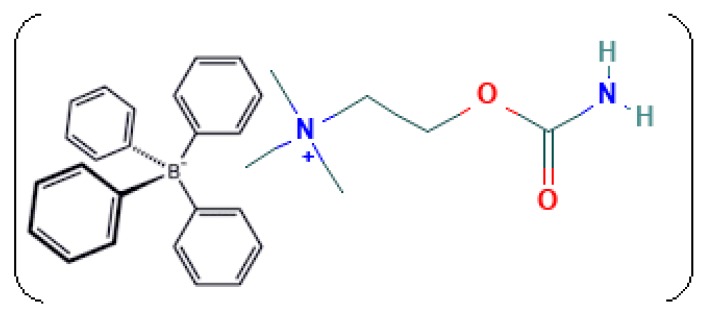
The proposed structural formula of the ion-association complex.

The present study originated from the fact that carbachol is a positively charged quaternary ammonium choline ester that acts as a cationic exchanger, which reacts with NaTPB as an anionic exchanger. It has been found that NaTPB is the optimum anionic exchanger due to its suitable grain size and low solubility. The proposed structural formula of the ion-association complex formed is shown in Figure 6.

The ratio between carbachol and the anionic exchanger was found to be 1:1, as proven by elemental analysis. The high selectivity of the method was achieved by utilizing this ion-association complex, and excellent selectivity coefficients were proven using the separate solution method.

The proposed sensors have the benefit of using nanocomposites, which are considered as excellent candidates for dielectric enhancement. Furthermore, graphene and MWCNTs have got interesting physicochemical properties, such as high surface area, high rigidity, high tensile strength, and high electrical conductivity [1]. In the present work, excellent carbachol recovery values were obtained in pure form, ophthalmic preparations, plasma, and urine using the proposed sensors. Analysis of carbachol in plasma and urine is of great importance in cases of carbachol oral intoxication. To the best of our knowledge, the only method presented in the literature had determined carbachol level in plasma and urine using liquid chromatography–tandem mass spectrometry along with cation-exchange column for solid-phase extraction of plasma samples [12]. In our simple, low-cost, and fast potentiometric method, we successfully analyzed carbachol in these matrices with no need for tedious extraction or pretreatment methods.

In addition, the proposed sensors were found to offer long lifetime as sensors 1 and 2 were stable for up to 1.5 and 2 months, respectively. Our method is also considered eco-friendly compared to other methods that use toxic hazardous VOCs. Analysis of carbachol in presence of varying concentration of its metabolite choline was successfully demonstrated with excellent recovery values.

Although graphene is reported to have a little bit more effectiveness in conductivity enhancement than carbon nanotubes [1], our study showed that the MWCNT-based sensor gave more accurate, precise, and selective results. It also showed longer lifetime. From our point of view, the reason behind this is most likely attributed to the fact that MWCNTs have the ability to promote a more effective electronic transfer. They are also more robust and elastomeric than graphene [7]. Moreover, the special structural properties lend MWCNTs some overwhelming advantages in fabricating electrochemical sensors, such as larger specific area, tubular nanostructure and electrocatalytic activity, thus resulting in high sensitivity, high strength, and chemical stability [42]. Furthermore, experimental studies have found that nanotubes are the stiffest and strongest fibers ever produced, which is obviously correlated with better mechanical and electronic properties of this material [43].

Both proposed sensors were also demonstrated as a promising and reliable tool for the determination of carbachol existing in ophthalmic preparations and for the rapid on-site analysis of carbachol levels in plasma and urine in cases of carbachol intoxication.

A comparison was carried out between our proposed method and the official method, and the results demonstrated that there was no significant difference between the proposed method and the official one.

## 5. Conclusions

In this work, we have demonstrated two nanocomposite-based sensors that can be applied for simple, rapid, and low-cost determination of carbachol in ophthalmic preparations, in presence of its metabolite choline, and in plasma and urine. The sensors gave precise and accurate results and can therefore be used for monitoring carbachol down to a concentration level of 1 × 10^−7^ M. Such sensors are very promising candidates as alternatives to sensors utilizing VOCs. They are simply and rapidly prepared using cheap, green, and nontoxic materials. Moreover, they offer the advantage of low LOD and high selectivity.

## Figures and Tables

**Table 1:** General electrochemical characteristics of the proposed sensors.

Parameter	Graphene Sensor 1	MWCNTs Sensor 2
Slope (mV/decade)	50.80	58.14
Intercept (mV)	487.27	605.48
LOD (M)	1.2 × 10^−8^	1.1 × 10^−8^
Response time (seconds)	5	5
Working pH range	4–8	4–8
Concentration range (M)	10^−7^–10^−2^	10^−7^–10^−2^
Stability (days)	45	60
Average recovery ^1^	98.68	100.41
Correlation coefficient	0.9992	0.9998
Intraday precision ^2^ (%RSD)	1.2	0.50
Interday precision ^2^ (%RSD)	2.1	0.11

^1^ Average of three determinations on six concentration levels. ^2^ Average of nine determinations.

**Table 2:** A comparison between the proposed sensors and the published electrochemical method [28].

Parameter	Proposed Nanoscale Sensors	Ordinary Scale Sensors [28]
LOD (M)	1.2 × 10^−8^–1.1 × 10^−8^	2.6 ×10^−^^6^–1.2 × 10^−^^7^
Response time (seconds)	5	6–8
Sensitivity (M)	1 × 10^−7^	10^−^^5^–10^−^^6^
Stability (days)	45–60	28–30
Average recovery ^1^	98.68–100.41	-
Intraday precision ^2^ (%RSD)	0.5–1.2	-
Interday precision ^2^ (%RSD)	0.11–2.10	-
Impact on environment	No VOC used	THF used
Applications	Plasma Urine Ophthalmic solutions	Ophthalmic solutions

^1^ Average of three determinations on six concentration levels. ^2^ Average of nine determinations.

**Table 3:** Potentiometric selectivity coefficients (Log K^pot^_carbachol, interferent_) for the proposed sensors.

Interferent	Log K for Sensor 1	Log K for Sensor 2
Choline	0.7 × 10^−3^	0.2 × 10^−4^
Benzalkonium chloride	0.3 × 10^−3^	0.3 × 10^−3^
NaCl	0.4 × 10^−3^	0.8 × 10^−4^
KCl	0.4 × 10^−3^	0.3 × 10^−4^
CaCl_2_ hydrate	0.4 × 10^−3^	0.3 × 10^−4^
2-chloroethanol	0.5 × 10^−3^	0.2 × 10^−3^
Urea	0.3 × 10^−3^	0.2 × 10^−4^
K carbamate	0.7 × 10^−3^	0.3 × 10^−3^
Triethylamine	0.4 × 10^−3^	0.8 × 10^−3^

**Table 4:** Application of proposed sensors in Miostat and Jestryl ophthalmic solutions.

Pharmaceutical Preparation	Sensor 1 Recovery% ± SD	Sensor 2 Recovery% ± SD
Miostat 0.01%	99.74 ± 2.11	100.98 ± 0.314
Jestryl 0.00025/mL	100.33 ± 1.815	100.07 ± 0.487

**Table 5:** Determination of carbachol in spiked human plasma and urine samples by the proposed sensors.

**Plasma Samples (Sensor 1)**			**Plasma Samples (Sensor 2)**	
**Claimed (M)**	**Found (M)**	**Recovery ^1^%**	**Found (M)**	**Recovery ^1^%**
1 × 10^−7^	1.04 × 10^−7^	104.00	9.94 × 10^−8^	99.40
1 × 10^−5^	9.87 × 10^−6^	98.70	9.98 × 10^−5^	99.80
1 × 10^−3^	1.02 × 10^−3^	102.00	9.98 × 10^−2^	99.80
Mean ± SD		101.60 ± 2.68		99.70 ± 0.23
**Urine Samples (Sensor 1)**			**Urine Samples (Sensor 1)**	
**Claimed (M)**	**Found (M)**	**Recovery ^1^%**	**Found (M)**	**Recovery ^1^%**
1 × 10^−7^	1.08 × 10^−7^	108.00	9.97 × 10^−8^	99.70
1 × 10^−6^	9.93 × 10^−7^	99.30	9.94 × 10^−7^	99.40
1 × 10^−5^	1.07 × 10^−5^	107.00	9.99 × 10^−6^	99.90
Mean ± SD		104.80 ± 4.76		99.70 ± 0.25

^1^ Average of three determinations.

**Table 6:** Determination of carbachol in presence of its degradation product, choline.

Choline Concentration	Sensor 1 Recovery %	Sensor 2 Recovery %
10.0%	99.59	100.11
20.0%	101.50	100.60
30.0%	100.23	101.62
40.0%	101.82	100.81
50.0%	101.21	100.03
60.0%	102.17	100.75
70.0%	102.73	101.46
80.0%	103.11	102.33
90.0%	102.92	102.34
Mean ± SD	101.70 ± 1.21	101.12 ± 0.87

**Table 7:** Statistical comparison between the determination of carbachol and the official method.

Parameter	Sensor 1	Sensor 2	The Official Method [32]
Mean %	98.68	100.41	99.72
SD	1.21	0.86	1.19
Variance	1.46	0.74	1.42
n	6	6	4
Students *t*-test	1.33 (2.36) *	−1.0 (2.57) *	--
F-test	1.03 (9.01) *	1.92 (5.40) *	--

* The figures in parenthesis are the corresponding theoretical values for F and t at *p* = 0.05.
